# Gunshot Injuries to the Eyes
1Read before the Medico-Chirurgical Society, December 15th.


**Published:** 1915-12

**Authors:** E. H. E. Stack

**Affiliations:** Capt., R.A.M.C.(T.F.); Specialist in Ophthalmology to the 2nd Southern General Hospital


### GUNSHOT INJURIES TO THE EYES.[1]

BY

E. H. E. Stack, M.B., F.R.C.S.,

*Capt., R.A.M.C.(T.F.); Specialist in Ophthalmology to the 2nd Southern General Hospital.*

### EYE INJURIES AT THE 2ND SOUTHERN GENERAL HOSPITAL.

We have to deal at this hospital with over one hundred cases a month of trouble of some sort connected with the eyes.

[1] Read before the Medico-Chirurgical Society, December 15th.

About one-third of these are due to injury or disease. An injury may affect the eye directly, or be the result of some cerebral trouble at a distance.

We have seldom had to trephine for injuries to the inner table, because experience has shown the necessity of doing this operation as soon as possible after the injury has been received, and there are therefore great numbers done in France on the first possible opportunity.

In cases of injury to the vault which have not been trephined, or in which, in spite of trephining, cerebral symptoms persist, then an œdema of the disc, which is very often present, is a marked extra indication for exploring. If in addition there is any contraction of the field of vision the indication is all the stronger, but when there is hernia cerebri or other signs of local sepsis, then it is just as well not to be in too great a hurry.

A man of 26 had a glancing wound of the right parietal bone with local suppuration and some necrosis of the outer table. For some weeks he was lethargic, slightly feverish, and had persistent œdema of both discs. On several occasions Colonel Michell Clarke suggested exploration, but I was loth to do this on account of the local infection. When the piece of bone exfoliated he improved, and was soon able to get about, but he had persisting tendency to slight giddiness. I don't know now whether I should have trephined or not.

From a scientific point of view it is a great pity that our patients have to be moved on so fast. The greater number of them one only sees a few times, and then they go, to be heard of no more; consequently one has but little opportunity of getting an idea of prognosis.

### I. SHELL SHOCK.

This is the most interesting eye problem which has arisen in the war, and the pathology is very difficult.

Several men at first sight seemed to be suffering from this condition, but when they came to be tested they got normal or nearly normal vision with glasses, usually hypermetropic astigmatism. Some were old blind eyes discovered for the first time, and possibly one or two were malingerers, although I never was able to prove this.

Then again men who had lost the sight of both eyes were usually sent to other hospitals, as we have had, as far as I know, only two cases of men blind in both eyes from any cause.

Many cases of blindness from "shell shock" get well rapidly. Putting all these facts together, it is not to be wondered at that we have had but few to illustrate this type of injury.

Private S. received no wound; a shell burst close in front of him, and he fell down. He noticed that at first he saw very badly, but soon recovered sufficiently to see pretty well with the left eye.

On admission all details normal except some photophobia in a bright light, and a little lachrymation in both eyes, no scotoma or Berlin dimness, the fundus being quite natural, and there was no error of refraction.

The vision in the right eye was $\frac{6}{60}$ and in the left $\frac{6}{5}$. Vision a week later in the right eye was much better, I think about $\frac{6}{12}$, but unfortunately I have omitted to note it. He showed no other signs of neurosis, and was quite sure that previously his vision in both eyes was good; he had always used his right eye for shooting.

Another case was complicated by having had epilepsy at rare intervals all his life. He was in a highly neurotic state, and when I first examined him he could only count fingers. However, a few minutes later he was persuaded to read $\frac{6}{5}$ with each eye, having two glasses in the frame, one being a $+1$ and the other a $-1$, which of course exactly neutralised each other.

I am very sceptical whether there is anything out of the ordinary in the cases who have developed defective vision after shell explosions. Either they are struck on the head without knowing it, or else they become for the time being subjects of hysteria or some other mental condition.

*Nystagmus.*—Quite a number of men have returned from the front suffering from this condition, which was not present before they went out.

I have seen about six of these. They are all of the same type, a coarse lateral nystagmus, rather worse when trying to fix an object, but very nearly constant. They are not made worse by looking up, as in miners. They show no evidence of intracranial pressure or of cerebellar disease. They are mostly of a neurotic type, and vision is a good deal interfered with.

For instance, a man whom I saw this morning, who up to the time he joined the army had been earning thirty-five shillings a week driving a tram. In France he fell on the top of his head and was stunned and cut slightly, but his wound was trifling. I saw him first nine months ago, and his condition is just the same now.

Coarse constant lateral nystagmus, fundus natural and vision *R.* and *L.* $\frac{6}{24}$, not improved by glasses.

This must be a purely cerebral condition, and I should think due to an actual lesion.

II. BLOWS ON THE EYES, INCLUDING BLOWS ON BONES IN THE NEIGHBOURHOOD, WHICH EXHIBIT NO SIGNS.

There is a history of the blow, and there is defective vision. These may be divided into two categories.

1. *Those which have an error of refraction.*—There have been quite a number of these, almost all hypermetropes.

Private E., aged 25, struck by head of a nail on the right eye, One week later tension −1, vision *R.* $\frac{6}{60}$. Fundus normal. Twelve days later vision

$$R.\ \frac{6}{36},\ \text{c.} + 2.5 = \frac{5}{5},\ L.\ \frac{5}{5},\ \text{c.} + 1.5 = \frac{5}{5}.$$

There have been many cases of this sort where, although no previous symptoms of eye-strain have been known, the blow has been the cause of sufficient interference with accommodation to bring out the latent hypermetropia. I expect that this paresis is only temporary.

2. *Those with no error of refraction.*

Private L., 4th Suffolks. Left eye bruised by a bullet. Ten days later vision $\frac{6}{36}$, no hypermetropia. He improved a good deal before discharge.

There have been several other cases identical with this one, and two at least recovered perfect vision.

### III. BLOWS ON THE EYE GIVING RISE TO PHYSICAL SIGNS.

1. *Photophobia and lachrymation.*—These have been very common, and often persist for much longer than one would expect, seeing that no obvious damage has been done.

2. *Subconjunctival hemorrhages* have often been noted, vision is generally much interfered with at first, there is but little conjunctivitis.

Private H., R.F.A., G.S.W. Two weeks ago sclerotic bruise. Vision, fingers at four yards, faint haze in the macular region, no definite scotoma could be made out.

Three weeks later vision $\frac{6}{36}$. The prognosis in this class is poor. Those who get well improve much more rapidly than this.

Private T., 2nd Essex. Struck ten days ago by a comrade's bayonet. Sclerotic hemorrhage in the right eye. Vision $\frac{1}{60}$. Twelve days later vision $\frac{6}{18}$. Fundus normal.

3. *Keratitis.*—Most of these are due to splashing of mud as the result of shell explosions, and there is nothing peculiar about them except, perhaps, that they take longer to get well than one would expect.

4. *Damaged iris and hyphæma.*—There have only been three or four of these.

Sergeant W., G.S.W. Torn and tucked-up iris, slight diplopia, and perception of light only; some vitreous hemorrhage with a red reflex; vision later $\frac{6}{60}$ c. $-2$, spherical, and a cyl $\frac{6}{9}$. Fundus seen a little foggy and disc natural.

5. *Traumatic cataract.*—There have been a good many of these, most of whom moved on before the question of operation arose, while the others, for some reason or other, were advised to postpone operation to a later date.

It is not a settled point when a traumatic cataract should be removed; some advise early excision instead of needling, claiming less liability to irremovable bits of capsule, while on the other hand some lenses become fluid, and can be almost completely evacuated through a small incision.

6. *Vitreous opacities.*—The prognosis is much worse as regards return of vision, but occasionally there is a much better result than one would expect.

Sergeant G., aged 29, was struck in the right eye by a piece of shrapnel. He did not notice anything for five weeks, and then muscæ appeared. On admission vision *R.* $\frac{6}{5}$ c. + .75 $\frac{6}{5}$. Retinoscopy in each eye showed between 4 and 5 D. of hypermetropia.

Ophthalmoscopic examination revealed extensive fine vitreous opacities without any change in the retina.

So that he had perfect vision in spite of a blow which caused hemorrhage into the vitreous.

Private G. had a rifle wound which fractured his right malar bone.

There was no red reflex obtainable except an occasionally-seen small red spot like a hole in a black curtain; with oblique illumination dark masses of clot could be seen moving behind the lens, which had not been damaged. I rather suspect a spicule of bone perforated the globe in this case, as the tension was low.

7. *Changes at the macula.*—Haab considers the macula as the most vulnerable point of the fundus.

Commotio retinæ is a term which should be confined to cases in which after a blow in the neighbourhood or on the globe there is œdema of the retina near the spot hit, or at the macula or at both.

This œdema is said to be due to vascular changes following the anæmia caused by the blow, though it is difficult to see how such a transient anæmia could give this effect.

This œdema at the macula may pass off in four or five days. Collected cases show that permanent central defective vision results in about a third of the cases.

Rarely retinitis proliferans follows, showing that at least in some there is retinal hemorrhage.

I have had only two cases showing this macular œdema. The first I have already mentioned under the head of sub-conjunctival hemorrhage. In it there was a faint whitish haze round a red macula and two or three radial striæ passing from this downwards, but even when his vision had improved to $\frac{6}{36}$ I could find no evidence of a central scotoma.

R., aged 25, 2nd Grenadier Guards, had been the crack shot of his regiment, using his right eye.

Ten days previous to admission a rifle bullet had turned up a flap of skin on the right eyebrow. He was not unconscious at the time, and when I saw him his vision in the right eye was reduced to perception of light. The disc was very white, but the arteries were not small; there was well-marked faint whitish œdema round the macula. He was in for several weeks without showing any improvement in his vision.

There have been no cases showing the so-called "hole in the macula."

8. *Ruptured choroid.*—I have seen only one of these, and it had two peculiarities. The man said he had not been struck, but that a shell had burst close in front of him. The rupture had the typical vertical crescentic appearance, but was situated to the temporal side of the macula, which was normal, instead of the usual situation between it and the disc. His vision was p. l. only.

9. *Foreign bodies in the eye.*—One situated in the lens was infected on admission, and the eye had to be removed.

Another was a small piece of shrapnel buried very deep in the cornea, where it had been for six weeks without giving much trouble, and scarcely interfering with vision.

With Mr. Richardson Cross's kind assistance I removed it with Haab's giant magnet at the Eye Hospital.

10. *Foreign bodies in the orbit.*—In one case I removed a piece of shell from the frontal sinus and left a larger bit in the sphenoidal sinus. He had some œdema of his disc, and very good vision. He went out healed without any symptoms.

11. *Ruptured globes* had almost all been enucleated before admission.

12. *Papillœdma.*—There have, of course, been numerous cases in this category, but I do not intend going through them, as they are better dealt with by the neurologist. One point noticeable about them is that many have, in addition to the swelling of the papilla, a ring of œdema in the retina surrounding the disc, the swelling is not often more than 2 D., and I have seen no case with retinal hemorrhages.

13. *Optic atrophy.*—There have been four of these. One certainly was due to damage or rupture of the optic nerve behind the eye.

14. There have been three cases of quadrantic scotoma due to wounds in the occipital region.

### IV. LACHRYMAL OBSTRUCTION.

Most of these are not due to injury, and occurred in troops in our neighbourhood who had not seen active service, but I should like to emphasise the good and rapid results I obtained by doing West's intranasal operation on the sac.

There is one man in the 2nd Southern General Hospital now who was under my care at Southmead last year. He came in with the first batch of wounded which we had.

A bullet had crossed his nose. The external wounds healed rapidly, and he was sent on Home Service duty.

He has had some necrosis of the ethmoid with the inferior turbinated bone adherent to the septum, and complete blockage of the tear duct. He will require an excision of the sac or a West's operation, if the nasal condition permits.

Plastic surgery of the lids has been frequently performed, but claims no special mention.

One man with a cut lower lid seemed much amused because a bullet had smashed his eye, which happened to be a glass one.

The three things which have struck me most with regard to these eye injuries have been, firstly, the number of glancing wounds of the eyeball, which, while causing considerable damage to the eye, have left untouched the surrounding soft parts; secondly, the frequency with which blows have brought to light for the first time quite marked degrees of hypermetropia; and, thirdly, the number of men who have acquired nystagmus as the result of head injuries.

